# A phase I study of BMS-690514 in Japanese patients with advanced or metastatic solid tumors

**DOI:** 10.1007/s00280-012-1932-9

**Published:** 2012-08-10

**Authors:** Hiroshi Nokihara, Noboru Yamamoto, Yasuhide Yamada, Kazuhiko Yamada, Taizo Hirata, Yasushi Goto, Maki Tanioka, Yoko Ikeda, Tomohide Tamura

**Affiliations:** 1 Internal Medicine and Thoracic Oncology, Division of Internal Medicine, National Cancer Center Hospital, 5-1-1, Tsukiji, Chuo-ku, Tokyo 104-0045 Japan; 2 Internal Medicine and Gastrointestinal Oncology, Division of Internal Medicine, National Cancer Center Hospital, 5-1-1, Tsukiji, Chuo-ku, Tokyo 104-0045 Japan; 3 Research and Development Division, Bristol-Myers K.K., 6-5-1, Nishi-shinjuku, Shinjuku-ku, Tokyo Japan

**Keywords:** BMS-690514, Tyrosine kinase inhibitor, Phase I trial, Solid tumor

## Abstract

**Purpose:**

BMS-690514 is a novel oral tyrosine kinase inhibitor of ErbB and vascular endothelial growth factor receptor. This open-label phase I dose-escalation study (ClinicalTrials.gov Identifier: NCT00516451) aimed to assess the safety, preliminary efficacy, pharmacokinetics, and pharmacodynamics of BMS-690514 in Japanese patients with advanced or metastatic solid tumors.

**Methods:**

Patients with advanced or metastatic solid tumors received oral BMS-690514 once daily continuously until disease progression or intolerable toxicity occurred. Dose-limiting toxicity (DLT) was evaluated from the first dose to Day 29. Dose levels at 100 and 200 mg were investigated. Assessments included adverse events, tumor response, pharmacokinetics, pharmacodynamics, 2 [18F] fluoro-2-deoxyglucose positron-emitting tomography, and epidermal growth factor receptor and K-ras mutations.

**Results:**

BMS-690514 at the dose of 100 mg (*n* = 3) or 200 mg (*n* = 3) was administered once daily to totally nine patients and was well tolerated up to 200 mg. No treatment-related serious adverse events or DLTs were reported. Frequently observed treatment-related AEs were acne, diarrhea, dry skin, hypertension, stomatitis, blood fibrinogen increased, hemoglobin decreased, pruritus, and hypoalbuminemia. These were generally reported as Grade 1 and 2. Five of 9 patients (56 %) had stable disease. Plasma concentrations of BMS-690514 reached Cmax within 3 h and declined with an effective half-life of approximately 10 and 12 h at 100 and 200 mg, respectively.

**Conclusions:**

Oral BMS-690514 was well tolerated in Japanese patients with advanced or metastatic solid tumors up to 200 mg.

## Introduction

Angiogenesis and tumorigenesis are complex processes in which tumors utilize multiple pathways to promote growth. Targeted inhibition of the vascular endothelial growth factor receptor (VEGFR) and other key signaling pathways (such as the epidermal growth factor receptor (EGFR) and HER2 pathways) has been clinically validated in the treatment for several solid tumor types [1–5]. Because inhibition of multiple pathways may provide synergistic antitumor effects, agents that target multiple pathways may be more effective than single-targeted agents. Moreover, agents that simultaneously inhibit VEGFR and EGFR or multiple members of the ErbB family may have the potential to overcome resistance associated with single-targeted treatments [6, 7].

BMS-690514 is a potent reversible oral tyrosine kinase inhibitor (TKI) of EGFR, HER2, HER4, and VEGFR1, -2 and -3, which has been shown to inhibit the proliferation of a broad range of lung, colon, gastric, and breast tumor cell lines [8, 9]. In vivo, BMS-690514 demonstrated antitumor activity in a number of tumor xenograft models in which tumor growth depends on EGFR or HER2 signaling [9].

In the phase I/II study, CA187002, BMS-690514 demonstrated evidence of antitumor activity and disease control in patients with non-small cell lung cancer (NSCLC). The maximum tolerated dose (MTD) among the North American and European patients was determined to be 200 mg daily. The dose-limiting toxicity (DLT) was diarrhea [10].

The phase I study, CA187006, was developed to assess the safety, preliminary efficacy, pharmacokinetics, and pharmacodynamics of BMS-690514 in Japanese patients with advanced or metastatic solid tumors. This is the first study to assess the safety profile of BMS-690514 in Japanese patients.

## Patients and methods

### Patients and eligibility

Eligible subjects were Japanese patients aged ≥20 years with a histologic or cytologic diagnosis of any advanced or metastatic solid tumor (non-hematologic malignancy) for whom standard of care was ineffective or inappropriate. Patients were required to have a life expectancy of at least 3 months and an Eastern Cooperative Oncology Group (ECOG) performance status (PS) of 0–1. Prior anticancer treatments such as chemotherapy, radiotherapy, hormonal, antibody-targeted therapy, or surgery were permitted, provided at least 4 weeks had elapsed since the last therapy (at least 6 weeks for nitrosoureas, mitomycin C, and liposomal doxorubicin). Any toxicity related to prior therapy must have returned to ≤Grade 1 with the exception of alopecia for eligibility into this trial.

Patients were excluded from the study if they had any of the following conditions: symptomatic central nervous system (CNS) metastasis or active CNS metastasis requiring steroids; a history of thromboembolic disease or bleeding diatheses (not including minor hemoptysis); gastrointestinal disease or surgery that could impact the absorption of the study drug or inability to swallow tablets; concomitant second malignancies unless a complete remission was achieved ≥5 years prior to study enrollment and no additional therapy was required or anticipated during the study period; current symptomatic cerebrovascular disease (or vascular lesions that may be fatal in case of rupture), uncontrolled or significant cardiovascular disease, significant tumor invasion of major blood vessels, uncontrolled peptic ulcer, and any other medical history or concurrent disease considered to make the subject inappropriate for this study as determined by the investigator.

Patients were also excluded if they had a history of treatment with BMS-690514, exposure to any investigational drug within 4 weeks of enrollment, or exposure to drugs generally accepted to have a risk of causing Torsade de Pointes.

All patients provided written informed consent to participate in this study. This study was conducted in accordance with the Declaration of Helsinki and the applicable guidelines on good clinical practice, and the protocol and the informed consent received institutional review board/independent ethics committee approval.

### Study design

This was an open-label, Phase I, dose-escalation study conducted at National Cancer Center Hospital, Tokyo, Japan. The primary objective was to identify the MTD of BMS-690514 administered orally, once daily to Japanese patients with advanced or metastatic solid tumors. Secondary objectives were to assess the safety of BMS-690514, to determine the antitumor activity observed with BMS-690514, to characterize the pharmacokinetic profile of BMS-690514, and to explore the pharmacodynamic profile of biologic response to BMS-690514.

### Drug administration and dose-escalation procedure

Eligible patients received oral BMS-690514 100 or 200 mg once daily continuously until disease progression or intolerable toxicity occurred.

Patients fasted for at least 1 h prior to drug administration and for at least 1 h post-dose. On days of frequent pharmacokinetic blood sampling (Days 1–8), patients fasted at least for 4 h prior to drug administration and at least for 2 h post-dose.

DLTs were evaluated from the initial dose until assessment on Day 29. DLTs were defined as any of the following toxicities judged to be at least possibly related to treatment with BMS-690514: ≥Grade 4 diarrhea or Grade 3 diarrhea lasting more than 24 h despite treatment with anti-diarrhea medication; ≥Grade 3 nausea, vomiting, or fatigue despite best supportive care; sustained or repeated blood pressure >150/110 mm Hg (more than 2 measurements taken at least 24 h apart) recurring despite treatment with anti-hypertensive medication; other Grade 3 or greater non-hematologic toxicity; any grade toxicity which, in the judgment of the investigator or sponsor, required removal from further study medication; delayed recovery from toxicity related to treatment with BMS-690514 that deferred retreatment for up to 7 days; an electrocardiograph (ECG) that demonstrated drug-related QTcF > 500 ms or QRS ≥ 50 % above baseline or absolute QRS ≥ 180 ms; Grade 4 neutropenia (ANC < 500 cells/mm^3^) or thrombocytopenia requiring a platelet transfusion.

Dose levels were escalated from 100 mg according to the typical 3+3 design.

### Safety assessments

Adverse events (AEs) were evaluated according to the Common Terminology Criteria for Adverse Events (CTCAE v 3.0) throughout the treatment period until a minimum of 28 days after the last dose, or until all drug-related AEs had recovered to baseline or deemed irreversible. Safety evaluations were based upon medical review of AEs and the results of clinical laboratory tests, vital sign measurements, echocardiograms, electrocardiograms, physical examination, ECOG PS, and chest X-ray/computed tomography (CT) scan.

### Pharmacokinetic analyses

Blood samples were collected for pharmacokinetic evaluation up to 24 h post-dose on Day 1 and Day 8, as well as pre-dose on Day 15 and Day 29. Pharmacokinetic parameters [Cmax, Cmin, Tmax, AUC (TAU), effective half-life (T-HALF) [11], and accumulation index (AI)] were derived from plasma concentration versus time for BMS-690514. The effective T-HALF is calculated from an AI and a dosing interval (TAU).

### Tumor response

Tumor response was determined for all patients with measurable lesions according to modified World Health Organization (WHO) tumor response criteria. Tumor measurements by CT, magnetic resonance imaging (MRI) were obtained at baseline and every 8 weeks thereafter.

### Pharmacodynamic activity biomarker and PET assessments

Plasma samples for pharmacodynamic analysis were collected on Days 1, 8, and 29, and analysis of soluble VEGFR2 (sVEGFR2) and collagen IV protein concentration was performed. The relative percent change from baseline (Day 1) for each protein concentration was calculated for each on-treatment specimen. Archived tumor tissue was studied for biomarkers of EGFR mutation status, EGFR copy number, and K-ras mutation status. 2 [18F] fluro-2-deoxyglucose positron-emitting tomography (^18^FDG-PET) scans were obtained at screening and after 6 weeks of therapy for all patients evaluable for standardized uptake value (SUV) measured in tumor tissue.

### Statistical methods

All patients who received study medication were included in the analysis of safety and efficacy. All statistical analyses were performed using SAS Version 8.2 (SAS Institute, Cary, North Carolina, USA). Summary statistics were employed in the analysis of AEs by System Organ Class (SOC), Preferred Term (PT) and cohort.

## Results

### Patient disposition and demographics

Nine patients were enrolled and treated in this study; 3 and 6 patients received 100 and 200 mg BMS-690514, respectively. Patient characteristics are shown in Table 1. All patients had metastatic lesions. Tumor types included 5 NSCLC (adenocarcinoma), 2 leiomyosarcoma, 1 colon cancer, and 1 thymic carcinoma. All patients had received previous treatment for their cancer (median number of chemotherapy regimens (range) was 2.5 [1–5]; *n* = 8). Two patients received prior VEGFR-targeted therapy and 3 received prior EGFR-targeted therapy.

**Table 1:** Patient disposition and baseline demographic characteristics

	100 mg	200 mg	Total
All enrolled	3	6	9
Treated	3	6	9
Gender			
Male	1	4	5
Female	2	2	4
Age (years)			
Median	66	57	57
Min–Max	50–71	33–65	33–71
Performance status (ECOG) *n*(%)			
0	1 (33)	3 (50)	4 (44)
1	2 (67)	3 (50)	5 (56)
Prior therapy *n*(%)			
Surgery	1 (33)	4 (67)	5 (56)
Radiotherapy	1 (33)	2 (33)	3 (33)
Hormonal, immunological, or biologic	2 (67)	4 (67)	6 (67)
Chemotherapy	3 (100)	5 (83)	8 (89)
1 regimen	0	1 (17)	1 (11)
2 regimens	1 (33)	3 (50)	4 (44)
3+ regimens	2 (67)	1 (17)	3 (33)

### Safety

No DLTs were reported in this study. Treatment-related AEs of interest are summarized in Table 2. Most AEs were mild in severity. Frequently observed treatment-related AEs (*n*) were acne (9), diarrhea (8), dry skin (7), hypertension (7), stomatitis (4), blood fibrinogen increased (4), hemoglobin decreased (4), pruritus (4), and hypoalbuminemia (4). These treatment-related AEs were generally Grade 1 or 2 with the exception of Grade 3 diarrhea observed in 1 patient in the 100 mg cohort. The diarrhea lasted less than 24 h and was not considered a DLT. One patient in the 100 mg cohort had treatment-related AEs leading to treatment discontinuation (Grade 2 fatigue and Grade 2 anorexia). No treatment-related serious adverse events (SAEs) were reported in this study. One patient in the 200 mg cohort experienced a SAE (Grade 3 pneumonia) that was considered not related to treatment. AE profiles were similar between the DLT evaluation period and the entire treatment period. The main reason for treatment discontinuation was disease progression (*n* = 6).

**Table 2:** Number of patients with treatment-related adverse events of interest

	100 mg (*n* = 3)^a^				200 mg (*n* = 6)^a^				Total (*n* = 9)
	Grade 1	Grade 2	Grade 3	Any grades	Grade 1	Grade 2	Grade 3	Any grades	Any grades
Gastrointestinal disorders									
Diarrhea	0	2	1	3 (100)	1	4	0	5 (83.3)	8 (88.9)
Vomiting	1	0	0	1 (33.3)	1	1	0	2 (33.3)	3 (33.3)
Skin and subcutaneous tissue disorders									
Acne	3	0	0	3 (100)	5	1	0	6 (100)	9 (100.0)
Dry skin	3	0	0	3 (100)	4	0	0	4 (66.7)	7 (77.8)
Pruritus	1	0	0	1 (33.3)	3	0	0	3 (50)	4 (44.4)
Rash	1	0	0	1 (33.3)	2	0	0	2 (33.3)	3 (33.3)
Exfoliative rash	1	0	0	1 (33.3)	0	0	0	0	1 (11.1)
Vascular disorders									
Hypertension	0	2	0	2 (67.7)	1	4	0	5 (83.3)	7 (77.8)
General disorders and administration site conditions									
Fatigue	0	1	0	1 (33.3)	2	0	0	2 (33.3)	3 (33.3)
Metabolism and nutrition disorders									
Hypokalemia	0	0	0	0	1	0	0	1 (16.7)	1 (11.1)

^a^There were no Grade 4 treatment-related adverse events observed

### Pharmacokinetics

**Fig. 1:**
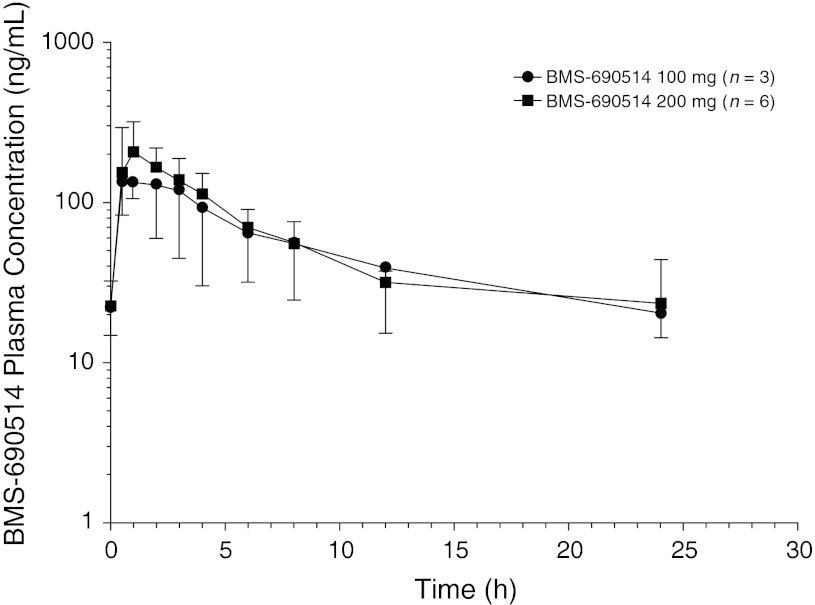
Plasma concentration-time profiles for BMS-690514 following once-daily oral doses of BMS-690514 to Japanese patients with solid tumors on Day 8

Following daily oral administration, plasma level of BMS-690514 reached peak concentration within 3 h and declined with an effective T-HALF of approximately 10 and 12 h at 100 and 200 mg, respectively (Fig. 1; Table 3). Both the geometric means in Cmax and AUC (TAU) were increased less than dose proportional (Table 3). Following repeated-dose administration, the pharmacokinetics did not change and the accumulation index values were 1.25 at 100 mg and 1.12 at 200 mg (Table 3). One patient was a homozygous, CYP2D6*10 genotype (intermediate metabolizer phenotype) and another was a homozygous UGT1A1*28 genotype (reduced expression phenotype). No association between natural variation in metabolizing enzyme genes and AUC (TAU) could be established.

**Table 3:**
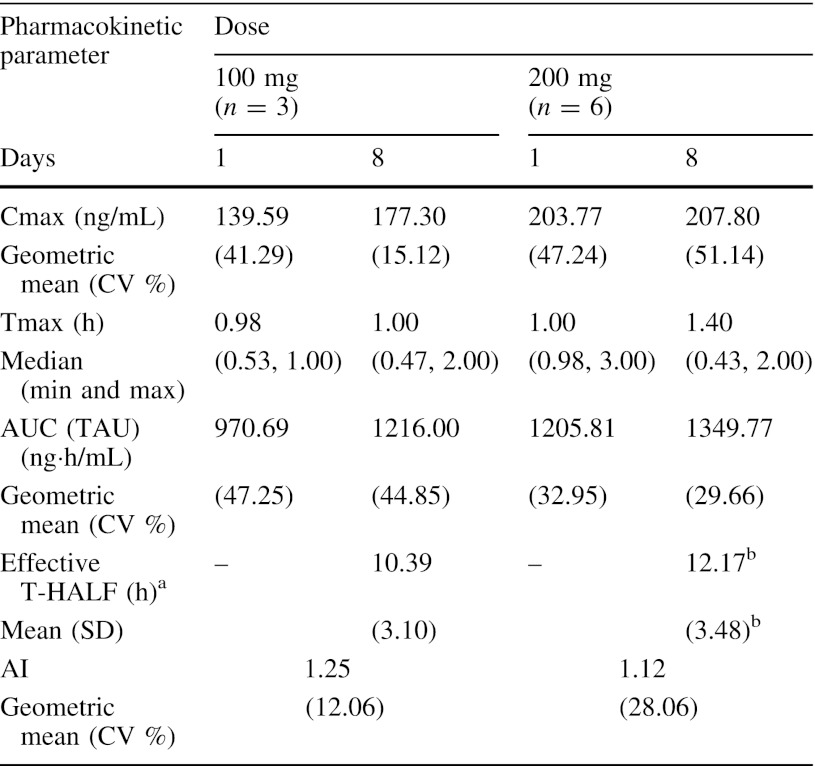
BMS-690514 pharmacokinetic parameters

^a^Effective T-HALF was calculated by the following formula, Effective T-HALF = TAU*ln (2)/ln[AI/(AI − 1)], where TAU is the dosing interval and AI is the ratio of AUC (TAU) on Day 8 to that on Day 1

^b^ *n* = 4

### Tumor response

Among the 9 patients evaluated in this study, 5 had stable disease (SD) and 4 had progressive disease (PD). Among the 5 patients with SD, 3 had NSCLC, 1 had leiomyosarcoma (on treatment for 6 months and stopped treatment), and 1 had thymic carcinoma. Time to progression was 3 months for 2 NSCLC patients. Among the 4 patients with PD, 2 had NSCLC, 1 had colon cancer, and 1 had leiomyosarcoma.

### Pharmacodynamic activity, biomarkers, and PET

**Fig. 2:**
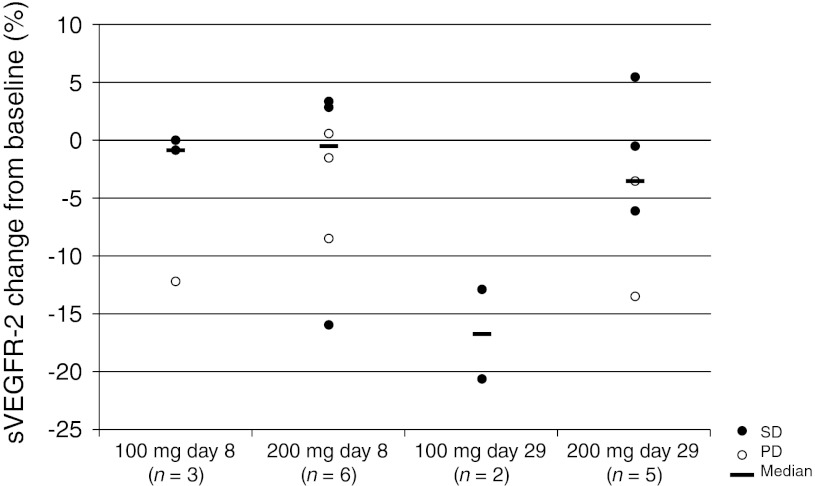
Percent change from baseline in sVEGFR2 after BMS-690514 administration

Relative percent changes from baseline for sVEGFR2 are presented in Fig. 2 for each patient. On Day 29, median changes were −17 and −4 % at 100 and 200 mg, respectively, with 6 of 7 patients showing at least a moderate decrease in sVEGFF2 regardless of dose. There was no apparent association between changes in sVEGFR2 protein and tumor responses to BMS-690514. No changes in collagen IV concentration were detected.

EGFR mutation, EGFR copy number, and K-ras mutation were assessed in 2 patients. The tumor sample from 1 NSCLC patient (SD) was EGFR-WT, with a normal EGFR copy number and K-ras mutation positive. The tumor sample from a patient with colon cancer (PD) was EGFR mutation positive, with a normal EGFR copy number and K-ras mutation positive.

^18^FDG-PET scan was completed for all patients. Of 5 patients with SD, 1 patient in the 100 mg cohort showed 25 % or more decreases in SUVmean, SUVpeak, and SUVmax. Another patient in the 100 mg cohort also showed 25 % or more decreases in the SUVmean and SUVmax and 23.4 % decrease in SUVpeak. No obvious correlation was detected between SUV parameters and exposure (AUC [TAU] on Day 8).

## Discussion

This was a phase I dose-escalation study in Japanese patients with advanced or metastatic solid tumors. The study was originally planned to determine MTD as the highest dose at which no more than one patient experience DLT out of 6 patients. Because the recommended phase 2 dose was determined at 200 mg in the global phase 1 study due to the toxic profile at 250 mg level, up to 6 patients was enrolled at 200 mg instead of dose escalation. The safety, efficacy, pharmacodynamics, and pharmacokinetics of BMS-690514 were explored. BMS-690514 once daily was well tolerated up to 200 mg. Although no DLTs were observed up to 200 mg dose, grade ≥2 diarrhea developed in 3 of 3 patients at 100 mg and in 4 of 6 patients at 200 mg. Drug-related AEs were mostly mild to moderate, and grade 3 diarrhea and anemia were observed in 1 patient in the 100 mg cohort. All AEs were reversible with medical management (early detection, monitoring, and adequate supportive care) or treatment interruption. The grade 3 diarrhea recovered to grade 2 or below within the onset day and was controlled by the administration of loperamide and *Bifidobacterium.*

The AEs reported for BMS-690514 are typical for TKIs that inhibit EGFR, HER2, or VEGFR2. Diarrhea and skin rash are considered class effects associated with EGFR inhibition [12], while hypertension, proteinuria, and bleeding events are well-established class effects of anti-VEGF therapy [13–15]. Importantly, the side effects observed with BMS-690514 did not appear to be greater than those observed with single-targeted therapies [14]. Therefore, BMS-690514 did not seem to have additive toxicity despite being a potent TKI of multiple signaling pathways.

Following oral administration, plasma levels of BMS-690514 rapidly increased, reaching peak concentration within 3 h and declined with an effective T-HALF of 10–12 h. The pharmacokinetics of BMS-690514 did not change during repeated administration, suggesting minimum accumulation of BMS-690514 in the plasma with once-daily dosing. There was no difference between the pharmacokinetic profile of BMS-690514 in this study and CA187002 study [10].

In total, of 9 treated patients, 7 had 2 or more prior chemotherapy regimens. Two patients had received prior VEGF-targeted therapy and 3 had received prior EGFR-targeted therapy. Despite the heavily pretreated status, overall best response of SD was achieved in 5 of 9 patients. Of special interest were 2 patients with NSCLC who were previously treated with gefitinib and who had SD on treatment with BMS-690514. Indeed, a previous clinical trial of BMS-690514 demonstrated similar antitumor and disease control activity in NSCLC patients previously treated with erlotinib. Here, mutation status was available in only 1 of the cases. SD was achieved in a subject with EGFR-WT and K-ras mutation-positive NSCLC. In light of evidence that BMS-690514 had disease control activity in patients whose tumors harboring the EGFR T790 M mutation associated with acquired resistance to EGFR-TKIs gefitinib and erlotinib [10], the current data are encouraging and suggest additional testing of BMS-690514 may be warranted, particularly in patients who have failed a prior EGFR TKI regimen.

Despite the limited number of patients in this study, there appeared to be a trend for sVEGFR2 to decrease from baseline by Day 29. Without considering treatment dose, the average decrease in sVEGFR2 over this period was 8 % for evaluable patients. This result coincides with the findings in the larger global phase I/II study [10], in which patients treated with the MTD showed an average Day 29 decrease in sVEGFR2 of approximately 8 %. Decreased levels of sVEGFR2 occurred using other VEGFR2 TKIs, including sunitinib [16], vatalanib (PTK787) [17], motesanib [18], cediranib [19], sorafenib [20], pazopanib [21], and axitinib [22]. While the process by which this change in sVEGFR2 occurs is not well understood, consistent decrease of sVEGFR2 has been seen as an excellent marker of pharmacological activity against the VEGFR. Therefore, VEGFR2 signaling in tumors is likely to be inhibited by BMS-690514.

A decrease in FDG uptake was observed in 2 patients with SD as measured by radiography. These data provide clinical evidence that BMS-690514 reduces tumor glucose metabolism and cell viability [23].

In conclusion, oral BMS-690514 was well tolerated in Japanese patients with advanced or metastatic solid tumors in doses up to 200 mg.
